# Most colorectal cancer survivors live a large proportion of their remaining life in good health

**DOI:** 10.1007/s10552-012-0010-2

**Published:** 2012-06-26

**Authors:** Isabelle Soerjomataram, Melissa S. Y. Thong, Majid Ezzati, Elizabeth B. Lamont, Wilma J. Nusselder, Lonneke V. van de Poll-Franse

**Affiliations:** 1 Department of Public Health, Erasmus MC, P.O. Box 2040, 3000 CA Rotterdam, The Netherlands; 2 Department of Global Health and Population, Harvard School of Public Health, Boston, MA USA; 3 Comprehensive Cancer Centre South, Eindhoven Cancer Registry, Eindhoven, The Netherlands; 4 Department of Medical Psychology, Center of Research on Psychology in Somatic Diseases (CoRPS), Tilburg University, Tilburg, The Netherlands; 5 Department of Epidemiology and Biostatistic, MRC-HPA Center for Environment and Health, School of Public Health, Imperial College, London, UK; 6 Department of Health Care Policy, Harvard Medical School, Boston, MA USA

**Keywords:** Colorectal cancer, Healthy life expectancy, Population-based, Survivors

## Abstract

**Purpose:**

Colorectal cancer (CRC) diagnosis reduces life expectancy and decreases patients’ well-being. We sought to assess the determinants of health and functional status and estimate the proportion of remaining life that CRC survivors would spend in good health.

**Methods:**

Using Sullivan method, healthy life expectancy was calculated based on survival data of 14,849 CRC survivors within a population-based cancer registry in southern Netherlands and quality of life information among a random sample of these survivors (*n* = 1,291).

**Results:**

Overall, albeit short life expectancy (LE at age 50 = 12 years for males and 13 years for females), most CRC survivors spent a large proportion of their remaining life in good health (74 and 77 %, for males and females, respectively). Long-term survivors may expect to live a normal life span (LE at age 50 = 30 years) and spent a large proportion of the remaining life in good health (78 %). In distinction, those with stage IV CRC had less than 2 years to live and spent more than half of their remaining life in poor health.

**Conclusions:**

Most CRC patients may expect no compromise on living a healthy life, underlining the importance of early detection. On the other hand, the high proportion of non-healthy years among stage IV CRC survivors confirms the importance of early detection and palliative care.

**Electronic supplementary material:**

The online version of this article (doi:10.1007/s10552-012-0010-2) contains supplementary material, which is available to authorized users.

## Introduction

Due to increased awareness of colorectal cancer (CRC) resulting in earlier detection and improvement in treatment, survival among CRC patients has been increasing [1]. Quality of life (QoL) among patients in the first 3 years after diagnosis is generally decreased, although it may improve with time since diagnosis [2]. Yet, many CRC survivors continue to live with long-term side effects of having had the cancer [3], especially related to its treatment. In the last decade, treatment for CRC has become more aggressive. It now includes new chemotherapy agents and combinations of agents, and new radiation and surgical approaches [4] for patients with both curable and incurable disease at the time of diagnosis. While these advances in treatment have been associated with increasing rates of survival, they are also associated with increasing rates of long-term side effects such as fatigue, gastrointestinal problems, urinary incontinence, and sexual dysfunction [4]. These side effects can be quite serious, aggravated by the fact that CRC survivors are generally older and commonly have other coexisting chronic diseases [5]. Besides physical discomfort, CRC also impacts psychologically on the patient, that is, fear of recurrence, anxiety, depression or negative body image which may lower quality of life [4, 6].

In this study, we determined the factors associated with self-reported health and physical functioning after CRC diagnosis using population-based data. This result was used to calculate the proportion of healthy life expectancy and disability-free life expectancy to be compared to that of the general Dutch population. Estimating the proportion of healthy life years is an innovative approach in oncological research and may serve as a tool to assess the level of cure among survivors not only by estimating survival but incorporating available information on survivors’ well-being and functional abilities. At the same time, it also detects groups with poor health state in need of extra care.

## Materials and methods

### Data

For the analyses, we used data of CRC survivors from the population-based Eindhoven Cancer Registry (ECR). The ECR records data of all newly diagnosed cancer cases in the southern of the Netherlands, an area with 2.4 million inhabitants [7]. The coverage area of the registry in the south of the Netherlands has gradually increased, covering about 0.9 million people in 1975 and over 2 million people since 1988. For all cancer patients, information on tumor stage at diagnosis (based on pathological and clinical tumor node metastasis (TNM) classified according to the UICC system [8]), type of primary treatment (type of surgery, chemotherapy, hormonal therapy, and radiotherapy), and comorbidity (collected since 1993) is routinely collected. Furthermore, age, sex, and socioeconomic class (based on zip codes, collected since 1983) [9] are registered.

Using the Dutch version of the SF-36 [10], QoL assessment was done in 2009 through postal questionnaires among a random sample of 1,692 CRC survivors (response rate 82 %) within the ECR [11, 12]. These survivors were diagnosed with CRC between 1998 and 2007. Medical specialists sent their (former) patients a letter to inform them about the study, together with the questionnaire. A reminder was sent if the questionnaire was not returned within 2 months. Approval for this study was obtained from a local certified Medical Ethics Committee.

### Analyses

We performed a multivariate logistic regression to assess the association of self-reported health and functional disability with clinical history. We adjusted for demographic factors such as gender, age (5-year age groups, and 85 years and over age group), and socioeconomic class: high, middle, low, and institutionalized (the later was excluded from further analysis due to low numbers). For clinical factors, we included subtype (colon and rectum), pathological staging (pTNM: I, II, III, and IV, 4 % unknown stage was excluded), treatment (surgery only, surgery with chemotherapy (high risk colon cancer stage II and all stage III [13]), and surgery with radiotherapy with or without chemotherapy (rectal cancer stage III [13])), comorbidity at diagnosis (none and one or more), and follow-up time (5 years or less and 6 years or more).

Using the survivorship history combined with the QoL assessment, we calculated healthy life expectancy (HLE) and disability-free life expectancy (DFLE). This calculation was only performed for the factors that influenced well-being or functional limitation as tested by the multivariate analysis. First, life expectancy was calculated by building life table based on the survival history of CRC survivors who were alive between 2005 and 2009. Data of the most recent five calendar years were used to ensure sufficient number of deaths and to have the most recent estimates for life expectancy. For the same reason, we included only data of survivors who were 50 or older at the time of the study. Finally, life expectancy was calculated based on data of 14,849 survivors. As for the health status, we used the QoL assessment of survivors older than 50 years at the time of the survey who responded to the question on general health (93 % of the total respondents, *n* = 1,291) and who answered the question on mobility limitation (88 % of the total respondents, *n* = 1,221). We dichotomized the 5-level answers of the SF-36 item “How do you rate your general health?” to excellent, very good and good = good, and fair and poor = poor. Because one may report good or healthy but have functional disability, we also included measure of daily functional ability from the SF-36 in our analysis. The prevalence of functional disability was calculated based on the 3-level answer to the SF-36 item “Are you limited to walk a few hundred meters due to your health condition?” We assigned functional limitation to those who answered “yes, very much limited” and “yes, a little bit limited.” Calculations were performed using the Sullivan method that accommodates cross-sectional information of the age-specific prevalence of general health status [14]. We calculated total and healthy life expectancies using the guide as suggested by the European Health Expectancy Monitoring Unit (EHEMU) [15]. Details of the calculation are presented at Appendix 1 (See online resources). All calculation was done using Stata 11.1 for Windows (StataCorp, Texas, USA).

## Results

**Fig. 1:**
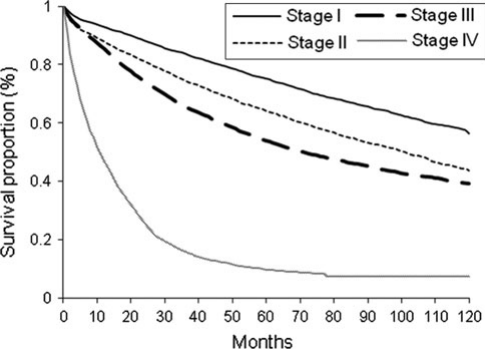
Survival proportion among colorectal cancer survivors, alive between 2000 and 2009

Between 2005 and 2009, 14,849 people aged 50 years and over were alive with history of CRC diagnosis in the ECR (53 % male, Table 1). Most survivors were 65 years or older (62 %) and commonly had other chronic illnesses at the time of diagnosis (63 % among those with known status of coexisting diseases). Two-thirds of the survivors were diagnosed with stage I or II CRC and had only surgical treatment. Five-year survival proportion by stage was shown in Fig. 1. There is a marked difference in survival by stage: 57 % of the cases with stage I survived at 10 years compared to 7 % of those with stage IV.

**Table 1:** Characteristics of living colorectal cancer survivors in 2005–2009

	Male (*n,* %)	Female (*n,* %)
*Age at diagnosis*		
<50 years	371 (5)	312 (4)
50–64 years	2,753 (35)	2,143 (31)
65–79 years	3,899 (50)	3,394 (48)
80 + years	800 (10)	1,177 (17)
*Age at in 2005*–*2009* ^a^		
50–64 years	2,329 (30)	1,712 (24)
65–79 years	4,127 (53)	3,423 (49)
80 + years	1,367 (17)	1,891 (27)
*Socioeconomic group*		
Low	1,725 (22)	1,970 (28)
Middle	3,042 (39)	2,556 (36)
High	2,531 (32)	1,884 (27)
Institutionalized	257 (3)	359 (5)
Missing	268 (3)	257 (4)
*Tumor site*		
Colon	4,731 (60)	4,875 (69)
Rectum	3,092 (40)	2,151 (31)
*Stage*		
I	2,121 (27)	1,763 (25)
II	2,697 (34)	2,587 (37)
III	1,875 (24)	1,784 (25)
IV	1,130 (14)	892 (13)
*Treatment*		
Surgery (S) only	4,321 (55)	4,525 (61)
S + chemotherapy	1,174 (15)	1,027 (14)
S + radio- ± chemotherapy	1,729 (22)	1,250 (17)
Other	599 (8)	635 (8)
*Comorbidity*		
0	2,353 (30)	2,239 (32)
1 or more	4,272 (55)	3,537 (50)
Unknown	1,198 (15)	1,250 (18)
*Total*	7,823	7,026

^a^This cohort (*n* = 14,849) was used to build the life table and calculate life expectancy. They were living colorectal cancer cases (CRC) in the year 2005–2009. These survivors were diagnosed with CRC between 1975 and 2009 in the catchments’ area of the southern Netherlands population cancer registry

### Determinants of poor health and physical disability

Poor health was associated with gender, socioeconomic status, tumor stage, and coexisting illnesses at diagnosis (Table 2). The strongest predictor of poor perceived health was stage. Survivors with stage IV were six times more likely to report poor health compared to those with stage I. Having one or more chronic disease at diagnosis was also one of the strongest predictors of poor health [Odds ratio (OR) = 2.5, 95 % confidence interval (95 % CI) = 1.8–3.4]. Low and middle socioeconomic class were also positively correlated with poor health [OR = 2.2 (1.5–3.1) and 1.5 (1.1–2.1), respectively]. Finally, female survivors were 50 % more likely to report poor health than males. Types of CRC treatment did not influence reported general health. If we did not include stage in the multivariate regression, the odds of reporting poor general health remained similar for those who had radiation with or without chemotherapy, increased to 1.1 (95 % CI: 0.8–1.6, from OR: 0.72) among those who had chemotherapy and to 5.4 (95 % CI: 1.4–20.1, from OR: 1.2) among those who had other therapy, respectively (results not shown).

**Table 2:** Perceived health in colorectal cancer survivors in 2009

	Good (*n*, %)	Poor (*n*, %)	Odd ratio^a^ (95 % CI) for poor health
*Sex*			
Male	561 (59)	169 (51)	1
Female	396 (41)	165 (49)	**1.49 (1.14–1.95)**
*Age at survey*			
50–64 years	289 (30)	88 (26)	1
65–79 years	551 (58)	181 (54)	0.98 (0.71–1.35)
80 + years	117 (12)	65 (19)	1.48 (0.96–2.27)
*Socioeconomic status*			
High	367 (38)	86 (26)	1
Middle	366 (38)	126 (38)	**1.49 (1.08–2.06)**
Low	178 (19)	102 (31)	**2.16 (1.51–3.10)**
Institutionalized	22 (2)	9 (3)	1.23 (0.51–2.96)
Unknown	24 (2)	11 (3)	1.80 (0.82–3.93)
*Tumor site*			
Colon	632 (66)	224 (67)	1
Rectum	325 (34)	110 (33)	1.24 (0.81–1.90)
*Time since diagnosis*			
≤5 years	722 (75)	263 (79)	1
6 + years	235 (25)	71 (21)	1.05 (0.76–1.45)
*Stage at diagnosis*			
I	270 (28)	97 (29)	1
II	385 (40)	112 (34)	0.77 (0.55–1.08)
III	272 (28)	87 (26)	1.11 (0.72–1.69)
IV	30 (3)	38 (11)	**5.76 (2.92–11.38)**
*Comorbidity at diagnosis*			
0	415 (43)	86 (26)	1
1 or more	459 (48)	231 (69)	**2.47 (1.82–3.35)**
Unknown	83 (9)	17 (5)	0.96 (0.53–1.73)
*Treatment*			
Surgery (S)	505 (53)	184 (55)	1
S + chemotherapy	200 (21)	68 (20)	0.79 (0.49–1.26)
S + radiotherapy ± chemotherapy	248 (26)	76 (23)	0.72 (0.45–1.14)
Other	4 (0.5)	6 (2)	1.22 (0.25–5.84)

Random sample of the colorectal cancer survivors within the catchment’s area of the registry in 2009 (*n* = 1,291)

^a^Multivariate analysis adjusted for sex, age, socioeconomic status, tumor localization, period of diagnosis, stage, comorbidity at diagnosis, colorectal cancer treatment

Bold value indicates* p* < 0.05

Similar results were observed for functional limitation (not being able to walk a few hundred meters, Table 3). In addition, we observed that being diagnosed with stage III CRC (besides stage IV) was also positively related to functional disability. Although not significantly related to perceived health, higher age was related to increasing functional limitation: CRC survivors aged 80 + years were four times more likely to have difficulty in walking a few hundred meters than those age 50–64 years.

**Table 3:** Functional disability (FD) in colorectal cancer survivors in 2009

	FD **−** (*n*, %)	FD **+** (*n*, %)	Odd ratio^a^ (95 % CI) for having FD
*Sex*			
Male	488 (59)	203 (52)	1
Female	342 (41)	188 (48)	**1.34 (1.03–1.74)**
*Age at survey*			
50–64 years	298 (36)	71 (18)	1
65–79 years	454 (55)	235 (60)	**2.03 (1.47–2.81)**
80 + years	78 (9)	85 (22)	**3.57 (2.30–5.55)**
*Socioeconomic status*			
High	339 (41)	91 (23)	1
Middle	320 (39)	145 (37)	**1.63 (1.19–2.24)**
Low	137 (17)	128 (33)	**2.77 (1.94–3.94)**
Institutionalized	14 (2)	14 (4)	**2.99 (1.30–6.85)**
Unknown	20 (2)	13 (3)	2.08 (0.96–4.48)
*Tumor site*			
Colon	540 (65)	275 (70)	1
Rectum	290 (35)	116 (30)	0.91 (0.59–1.42)
*Time since diagnosis*			
≤5 years	627 (76)	309 (79)	1
6 + years	203 (24)	82 (21)	0.98 (0.71–1.35)
*Stage at diagnosis*			
I	247 (30)	99 (25)	1
II	312 (38)	154 (39)	1.20 (0.87–1.67)
III	236 (28)	107 (27)	**1.61 (1.05–2.45)**
IV	35 (4)	31 (8)	**4.14 (2.04**–**8.39)**
*Comorbidity at diagnosis*			
0	372 (45)	101 (26)	1
1 or more	389 (47)	265 (68)	**2.10 (1.57**–**2.81)**
Unknown	69 (8)	25 (6)	1.22 (0.72–2.09)
*Treatment*			
Surgery (S)	420 (51)	227 (58)	1
S + chemotherapy	186 (22)	71 (18)	1.00 (0.61–1.62)
S + radiotherapy ± chemotherapy	220 (27)	87 (22)	0.64 (0.40–1.00)
Others	4 (0.5)	6 (2)	1.87 (0.38–9.24)

Functional disability was defined as limited to walk a few hundred meters

Random sample of the colorectal cancer survivors within the catchment’s area of the registry in 2009, 1,221 responded to the question of this particular outcome

^a^Multivariate analysis adjusted for sex, age, socioeconomic status, tumor localization, period of diagnosis, stage, comorbidity at diagnosis, colorectal cancer treatment

Bold value indicates* p* < 0.05

### Healthy life expectancy (HLE) and disability-free life expectancy (DFLE)

On average, a 50-year-old CRC survivor may live for another 12 years (men) and 13 years (women), with most of their remaining life spent in good health (77 % in men and 74 % in women, Table 4). Stage greatly determined life expectancy and the proportion of life years spent in good health. On the other hand, although there was no association between follow-up time and health status, long-term survivors, that is, followed for 6 years or more had a longer life expectancy as compared to short-term survivors, that is, followed for 5 years or less (life expectancy at 50 were 29.9 vs. 7.5 years, respectively). A marked difference was also observed between stage groups for example those diagnosed with stage I lived on average for another 25–30 years as compared to 2 years for those with stage IV. In addition, stage IV CRC survivors also had the shortest portion of remaining life in good health (47 % for men and 40 % for women). Furthermore, men with stage I–III colorectal spent 76–83 % of their life after cancer diagnosis without disability compared to only 53 % for those with stage IV.

**Table 4:** Life expectancy (LE, in years) healthy life expectancy (HLE, in years) and disability-free life expectancy (DFLE) among colorectal cancer survivors

	Men			Women		
	LE, years	HLE, years ( %)	DFLE, years ( %)	LE, years	HLE, years ( %)	DFLE, years ( %)
*Age* ^a^						
At age 50	12.3	9.5 (77)	9.5 (77)	13.3	9.9 (74)	9.8 (74)
At age 65	9.4	7.3 (77)	6.3 (67)	11.5	7.9 (68)	6.6 (58)
At age 80	5.5	3.8 (69)	3.2 (58)	7.0	4.1 (59)	2.6 (37)
*Socioeconomic status* ^a, b^						
High	12.5	10.0 (80)	10.2 (82)	14.0	11.8 (79)	11.2 (80)
Middle	13.7	10.9 (80)	10.8 (79)	13.3	9.9 (77)	10.2 (77)
Low	9.3	6.0 (65)	6.3 (68)	12.1	6.5 (56)	6.8 (56)
*Time since diagnosis* ^a, b, c^						
≤5 years	7.5	5.6 (74)	6.0 (79)	7.6	5.6 (74)	5.7 (76)
6 + years	27.6	22.3 (81)	20.8 (75)	32.7	24.5 (75)	22.6 (69)
*Stage* ^b^						
Stage I	25.3	19.1 (75)	19.2 (76)	29.8	21.1 (71)	21.3 (72)
Stage II	19.2	16.0 (83)	14.5 (76)	20.9	15.8 (75)	15.4 (74)
Stage III	13.6	10.7 (79)	11.3 (83)	16.8	12.9 (77)	11.9 (71)
Stage IV	2.1	1.0 (47)	1.1 (53)	2.2	0.9 (40)	1.2 (53)
*Comorbidity* ^a, b^						
0	12.8	10.4 (81)	10.6 (83)	14.8	14.8 (84)	12.2 (82)
1 or more	11.0	7.8 (70)	7.9 (72)	12.5	12.5 (62)	8.5 (66)

^a^The cohort contributed to 4,721 deaths with a total of 44,139 person years of follow-up (mortality rate of 106 per 10,000)

^b^Life expectancy at age 50

^c^Though follow-up time was not related to well-being and functional limitation, we calculated HLE and DLFE because of its relevance to proportion cured

From the other prognostic factors, two patterns emerged. First, groups who lived a larger part of their remaining life in poor health. This pattern was shown in female of the low socioeconomic class who could expect to live longer than their male counterpart (12 vs. 9.9 years) but spent 44 % of their remaining life in poor health (vs. men low socioeconomic class, 35 % in poor health). Another example was elderly female survivors (80 + years) who may expect to live as long as male survivors but would spent 41 and 63 % of their remaining life in poor health and with disability (vs. male, 31 and 42 %, respectively). Second, those who lived a long life with large proportion spent in good health, for example survivors from high socioeconomic status and those without comorbid disease at diagnosis. On average, male CRC survivors of high socioeconomic status may live for another 12.9 years at age 50 and spend 80 % of it in healthy life (vs. 9.9 years and 65 % among survivors from low socioeconomic).

## Discussion

On average, a 50-year-old CRC survivor might live ¾ of his/her remaining life in good health and without functional limitation. Being diagnosed with stage IV CRC is the most important predictor of poor perceived health and functional disability. Those diagnosed with advanced stage of CRC had a remarkably low life expectancy and showed a markedly lower proportion of HLE by spending half of their remaining life with disability. As for patients with stage I, II, or III CRC, they reported similar perceived health, though those with stage III were more likely to report functional limitation. Other vulnerable groups included survivors with one or more coexisting disease, women especially among the oldest old, and those from the lowest socioeconomic class.

### Study implication

#### Comparison to general population

CRC survivors had a much lower life expectancy as compared to the general Dutch population (12–13 years in our cohort vs. 30–34 years in the population) [16]. Yet, it seems that cure is reached among the long-term survivors as life expectancy similar to that of the general Dutch population, confirming results from conditional survival studies [17]. Another good news is that a large proportion of the remaining life years among survivors were spent in good health. This is reflected in studies on quality of life among CRC survivors that have reported good quality of life [2, 6] and similar physical limitation as the general population [4]. As compared to the Dutch population, CRC survivors spent even a larger proportion of life after cancer diagnosis in good health. At age 50, an average Dutch men would spend 66 % (vs. 77 % in survivors) of their remaining life in good health and Dutch women about 65 % (vs. 74 % in survivors) [18]. One explanation of this finding could be survival selection. Those who survived cancer are probably the strongest in the population, thus also having better general health. Differential in assignment of good or poor health may also explain the difference. Dutch statistics dichotomized good and very good health into healthy, and those who responded to general health question as average, fair, and poor were grouped into not healthy. In our questionnaires, only those who responded fair and poor were included in the non-healthy group because we did not have average as a choice of response. Third, this may be also caused by the non-response: 17 % of the survivors did not respond to our questionnaires. Though this is a relatively small number compared to other questionnaires-based study, nevertheless, non-respondents probably consisted of mostly those with worse health state [19]. Finally, the explanation for this difference (lower proportion of HLE among general population as compared to cancer survivors) could be the positive attitudes that cancer survivors adopt after cancer experience, a process which is referred to as “reframing” [20–22].

#### Vulnerable groups

A group who had the shortest life span and spent the largest proportion of their remaining life in ill health was survivors with stage IV CRC. This finding is in line with other studies that have indicated lower quality of life among patients with advanced CRC [2]. The true situation is probably worse because non-response was highest among those with stage IV causing underestimation in our estimate [19]. Physiologic burden from more aggressive and incurable cancer and its treatment has been suggested as cause of poor quality of life among these patients. Our findings underline the importance of early detection and palliative care.

We did not find an association between various treatments and self-perceived health or functional disability after correction for observable confounders. In the last few years, new treatment regimens have been employed on CRC patients. Because these treatments generally have higher side effects, we expected some impact on quality of life as compared to those who only received surgical treatment, especially among the elderly [5, 23]. Yet, treatment for CRC is largely determined by the stage of cancer at the presentation of the disease, making it difficult to disentangle their independent effect [13]. In a larger cohort within stage difference in assessing the impact of treatment on quality of life correcting for other confounders (incl. risk profile) should be done.

Other groups that showed higher likelihood of poor perceived health and disability with lower proportion of remaining life in good health were CRC survivors with comorbidity at diagnosis. Patients with comorbidity were probably more likely to be smokers or former smokers, overweight and physically inactive [24], and this might relate to the generally poor health status. Women aged 80 or older and of the lowest socioeconomic class had also a low proportion of HLE and DFLE compared to men from the same group. This is concurrent to findings from other studies [25]. that hypothesized the relation to the worse social and psychological factors among the elderly and women [25] Therefore, these groups might benefit from healthier lifestyle, better health care, and social support.

### Considerations in interpretation, limitations, and strength

Life expectancies reported in this study need to be interpreted with caution. It should not be extrapolated as the numbers of years left to live for CRC patients in general. The survivors that formed the cohort in this study consisted of mainly patients who were diagnosed in the study period: year 2005–2009 (49 %, whereas 33 and 21 % were diagnosed in 1998–2004 and 1975–1997, respectively). Mortality early after cancer diagnosis is high [17]. As seen in our analysis, those followed only for 5 years had a substantial shorter life expectancy as those who had a longer follow-up. In our sensitivity analysis (Appendix 2), we showed that including patients with longer follow-up does not change our final conclusion. In addition, we also included patients in terminal stage who contributed to the very high mortality and very few person years (see Fig. 1). Although these patients only contributed to 6 % of the total person years in the cohort, they contributed to 27 % of the total deaths. These two factors mentioned above account for the generally low life expectancy (when all stages were aggregated in a single life expectancy estimate) reported in our study.

Several limitations should be taken into account after reading our findings. First, our result relied solely on direct assessments of health states measured with questionnaires. This approach assumes that individuals use the same response thresholds in reporting [26]. However, following a cancer diagnosis, patients experience a dramatic change that may alter their internal values, leading to a shift in response for the assessment of health [27]. So the group that was expected to have poor health or disability reported similar or better health than the group who is expected to be better off. Finally, we only used three items from the whole domains in quality of life in measuring our outcome. In Appendix 3, we assessed the impact of including emotional domains into the analysis which resulted in similar finding to our main analysis. Earlier studies have shown, although having comparable QoL, colorectal cancer is experiencing long-lasting specific symptoms such as fatigue, nausea/vomiting, pain, insomnia, and appetite loss [28]. Smaller subgroups of patients, for example those living with stoma have also reported poorer quality of life [12]. Thus, limitations in these other areas could have been masked.

By using a population-based data, we ensured completeness and also reliability in the construction of the life table and its result. In this study, we incorporated patients’ survival and quality of life information in one estimate (HLE and DFLE). Assessment of patients’ prognosis after cancer diagnosis had mostly been based either on survival time or on quality of life. Having them both in a single measure will ease the valuation of life expectancy after diagnosis and useful for patients’ information or guidelines in clinical decision.

## Conclusions

Our finding suggests high proportion of healthy life expectancy among CRC survivors. All the more, for some groups of CRC survivors, the proportion of HLE exceeds that of the general population, highlighting the importance of early detection. Survivors of stage IV CRC are most likely to report poor health and spent a large proportion of their remaining life in ill health, highlighting the importance of early detection and palliative care. Survivors with comorbidity, elderly, women, and lower socioeconomic class also reported high prevalence of poor health and disability. This highlights the value of healthier lifestyle, and improvement in social and health care policies.

## Electronic supplementary material

Below is the link to the electronic supplementary material.

Supplementary material 1 (DOCX 18 kb)
