# Supplementary material for: Most colorectal cancer survivors live a large proportion of their remaining life in good health
Source: Cancer Causes Control. 2012 Jun 26;23(9):1421–8. doi: 10.1007/s10552-012-0010-2 (PMC3415614; doi:10.1007/s10552-012-0010-2)
Supplement: Supplementary file 1 — Supplementary material 1 (DOCX 18 kb) [file 10552_2012_10_MOESM1_ESM.docx]

**Appendix 1 Calculating healthy life expectancy (online resources)**

The calculation was done in two steps: first we built period life tables according to each variable that we were interested to examine, which provided us with specific life expectancy values. The calculation mainly required amount of person years and number of deaths in each age group. Because we included survivors within a 5-year period (2005-2009, which is necessary to be able to build multiple life tables according to various categories i.e. stage), person years were calculated annually to adjust for aging of the cohort. For example a patient who was diagnosed with CRC cancer at age 48 in 2004 and died in 2006 would not contribute person year in year 2005 (because he/she was only 49 years old), but did so in year 2006 for age 50. A patient diagnosed at age 53 in 2007 and survived until the end of the study (2009) contributed person years in 2007, 2008, 2009, for age 53, 54 and 55. Based on these person years and number of death, mortality probability was calculated and applied to a hypothetical cohort of 100,000 50 year olds. This was followed by the calculation of total number of years the cohort has yet to live. For age 50 we acquired the remaining life expectancy for at age 50 by dividing this total number of years the cohort has yet to live by the number of people still alive at age 50. The second step was to calculate the years spend in good health (HLE) and without functional limitation (DFLE). Here the prevalence data (health status) by age was multiplied with the total number of person years in each age group to divide the person years into healthy or unhealthy years. The health expectancy (HLE and DFLE) was thereafter calculated by summing the number of healthy person years over all age groups 50 and over and dividing the total number of healthy years by the number of people alive at age 50. Complete mathematical formulas can be found www.ehemu.com (reference: (15))

**Appendix 2 Sensitivity analysis (online resources)**

The population that was used to calculate life expectancy was from a different cohort (diagnosed with colorectal cancer between 1975-2009 who were alive between 2005 and 2009) than the cohort that contributed to the data on general health and functional limitation (diagnosed between 1998 and 2007 who were alive in 2009). Therefore in this sensitivity analysis, we calculated HLE and DFLE if life expectancy calculation was calculated based from the same cohort (colorectal cancer survivors who were alive in 2009 and were diagnosed with colorectal cancer between 1998 and 2007). Because only patients with 1 year or longer survival was included i.e. there was a 1-year gap since last diagnosis period (2007) and follow-up (2009), we expected that life expectancy would be longer. However proportion of HLE and DFLE would be similar to that found in the main analysis.

### Results

If we used survivors data who were alive in 2009 and diagnosed with colorectal cancer between 1998 and 2007, we had 275 deaths among males with 3029 person-years and, 191 deaths and 2773 person-years in females. In total mortality rates were 80 per 1000 (vs.106 per 1000 for the main analysis) As expected, life expectancy was higher than that was found for the main analysis, 13.8 and 17.8 years for males and females, respectively (vs. 12.3 and 13.3 years in the main analysis). HLE and DFLE proportions were the same as that found in the main analysis. The proportions of remaining life spent in good health were 77% and 73% for males and females, respectively.

**Appendix 3 Healthy Life Expectancy using emotional or physiological scale (online resources)**

Most colorectal cancer survivors reported comparable health as controls without history of cancer. Yet, they experience long-lasting detriments in specific symptoms(28) e.g. fatigue which may decrease emotional or psychosocial well-being. Reframing which is thought as part of the patients’ adaptation to their disease and its treatment(22) might explain this finding. As such, we expanded our analysis by reassessing the healthy life expectancy using emotional domain of the SF36 in the analysis, and we refer to this as the emotional Healthy Life Expectancy (eHLE). The prevalence of deficit in emotional/psychological domain was calculated based on the 2 levels answer to the SF-36 item “During the past 4 weeks, have you had any of the following problems with your work or other regular daily activities as a result of any emotional problems (such as feeling depressed or anxious)?” This item was further detailed into three items of activities and we used the first for our analysis (Cut down the amount of time you spent on work or other activities). Using the other two detailed items (a. Accomplished less than you would like, or b. Did not do work or other activities as carefully as usual) resulted in similar result as if we were to use the first item.

### Results

Similar as to our main analysis, colorectal cancer survivors spent most of their remaining life in good psychological health. On average, a 50 years old CRC survivor spent 84% (in men) and 83% (in women) of their remaining life in good emotional health (Table).

**Table: Life Expectancy (LE, in years) and emotionally Healthy Life Expectancy (eHLE, in years) among colorectal cancer survivors**

|  | Men | | Women | |
| --- | --- | --- | --- | --- |
|  | LE, years | eHLE, years (%) | LE, years | eHLE, years (%) |
| Age |  |  |  |  |
| At age 50 | 12.3 | 10.3 (84) | 13.3 | 11.0 (83) |
| At age 65 | 9.4 | 7.8 (83) | 11.5 | 8.7 (76) |
| At age 80 | 5.5 | 4.1 (74) | 7.0 | 5.0 (72) |
|  |  |  |  |  |
| Socioeconomic status ^a^ |  |  |  |  |
| High | 12.5 | 11.4 (91) | 14.0 | 12.2 (87) |
| Middle | 13.7 | 11.2 (82) | 13.3 | 10.6 (80) |
| Low | 9.3 | 7.0 (75) | 12.1 | 10.1 (86) |
|  |  |  |  |  |
| Time since diagnosis ^a^ |  |  |  |  |
| ≤5 years | 7.5 | 6.1 (81) | 7.6 | 6.5 (86) |
| 6+ years | 27.6 | 25.7 (93) | 32.7 | 25.2 (77) |
|  |  |  |  |  |
| Stage ^a^ |  |  |  |  |
| Stage I | 25.3 | 21.3 (84) | 29.8 | 25.0 (84) |
| Stage II | 19.2 | 16.6 (87) | 20.9 | 16.5 (79) |
| Stage III | 13.6 | 11.5 (84) | 16.8 | 13.8 (82) |
| Stage IV | 2.1 | 1.3 (61) | 2.2 | 1.8 (82) |
|  |  |  |  |  |
| Comorbidity ^a^ |  |  |  |  |
| 0 | 12.8 | 11.0 (86) | 14.8 | 12.9 (87) |
| 1 or more | 11.0 | 9.2 (83) | 12.5 | 9.9 (79) |
| ^a^ Life expectancy at age 50 | | | | |
